# The complete chloroplast genome of *Mahonia eurybracteata* subsp. *Ganpinensis* (H.Lév.) T. S. Ying & Boufford (Berberidaceae)

**DOI:** 10.1080/23802359.2019.1687346

**Published:** 2019-11-12

**Authors:** Ruoqi Huang, Caihong Wang, Qiong Liang, Ying Wang, Tae-Jin Yang, Yanjun Zhang

**Affiliations:** a Key Laboratory of Plant Germplasm Enhancement and Specialty Agriculture, Wuhan Botanical Garden, Chinese Academy of Sciences, Wuhan, China;; b University of Chinese Academy of Sciences, Beijing, China;; c Key Laboratory of Plant Resources Conservation and Sustainable Utilization, South China Botanical Garden, Chinese Academy of Sciences, Guangzhou, China;; d Guangdong Provincial Key Laboratory of Applied Botany, South China Botanical Garden, Chinese Academy of Sciences, Guangzhou, China;; e Department of Plant Science, Plant Genomics and Breeding Institute, Research Institute of Agriculture and Life Sciences, College of Agriculture and Life Sciences, Seoul National University, Seoul, Republic of Korea

**Keywords:** Chloroplast, genome sequence, *Mahonia eurybracteata* subsp. *ganpinensis*, phylogenetic relationships

## Abstract

*Mahonia eurybracteata* subsp. *ganpinensis* (H.Lév.) T.S.Ying & Boufford. is an evergreen shrub of Berberidaceae and has the potentials for horticultural and medicinal development. In the present paper, the complete chloroplast genome of *Mahonia eurybracteata* subsp. *ganpinensis* (H.Lév.) T.S.Ying & Boufford. was sequenced. The complete chloroplast genome was 165,562 bp in length, containing a large single copy region (73,394 bp), a small single copy region (18,698 bp) and two inverted repeat regions (36,735 bp). The genome consisted of 113 genes, including 79 protein-coding genes, 30 tRNA genes and 4 rRNA genes. Phylogenetic analysis showed that *M. eurybracteata* subsp. *ganpinensis* and *M. bealei* were firstly clustered into a branch and the two *Mahonia* species were most closely related to the genus *Berberis* of Berberidaceae.

*Mahonia eurybracteata* subsp. *ganpinensis* (H.Lév.) T.S.Ying & Boufford. is an evergreen shrub of Berberidaceae. The subspecies is mainly distributed in Guizhou, Sichuan, and Hubei of China (Zhang 1998; Ying et al. 2011). As evergreen shrubs or small trees, the plants of the genus *Mahonia* often used as horticultural ornamental plant (Jiang et al. 2007). Furthermore, the leaves, roots, stems, and barks of the plants of *Mahonia* have been reported with antibacterial, antifungal, anticancer, and anti-inflammatory effects (Ji et al. 2000; Ouyang et al. 2012; Latha et al. 2019). *Mahonia eurybracteata* subsp. *ganpinensis* has the potentials for horticultural and medicinal development.

The genus *Mahonia* comprises about 60 species of which about 30 are distributed in China. Although the researchers conducted molecular phylogenetic studies on *Mahonia* using ITS (Kim, Kim, Landrum 2004), there are quite a few questions on the infra-genera relationships of the genus. The chloroplast genome could provide valuable information for botanic taxonomy and phylogeny (Ma et al. 2013; Zhang et al. 2016), also basic genetic resource which are of implication for the phylogenetic studies.

The chloroplast DNA of *M. eurybracteata* subsp. *ganpinensis* was extracted from its fresh leaves which were sampled in Wuhan (N30°32′38′′, E114°24′51′′). The voucher herbarium specimen was deposited at the Herbaria of Wuhan Botanical Garden, Chinese Academy of Sciences (HIB) and the specimen Accession number is Yanjun Zhang 555 (HIB). A chloroplast genomic library was constructed with PCR technology and sequenced with Illumina Hiseq 2000 (Kim et al. 2017). Raw reads were screened with NGS QC toolkit software (Cai et al. 2015) to obtain high-quality reads which were spliced with CLC-quality genome assembler (ver 4.06bata) and MUMmer (Kurtz et al. 2004) by referring to the chloroplast genome of *Mahonia bealei* (Fort.) Carr. (KF176554). Gene annotation was done by using DOGMA (http://phylocluster.biosci.utexas.edu/dogma/) combined with the online alignment tool Blastx and ORF Finder (http://www.ncbi.nlm.nih.gov/). The tRNA genes were predicted by using DOGMA and the online sites tRNA-scan, ARAGORN (Laslett and Canback 2004). The circular cp genome map was finished by using the Orgnellar Genome DRAW (http://ogdraw.mpimp-golm.mpg.de/) (Lohse et al. 2007).

The chloroplast genome sequence of the *M. eurybracteata* subsp. *ganpinensis* was submitted to NCBI, and the accession number is MN417307. The genome sequence has a total length of 165,562 bp and the structure is a typical quadripartite, including a large single-copy region or LSC (73,394 bp), a small single copy region or SSC (18,698 bp) and two inverted repeat regions or IRs (36,735 bp). The GC contents were 38.07%. The chloroplast genome of *M. eurybracteata* subsp. *ganpinensis* consists of 113 genes, including 79 protein-coding genes, 30 tRNA genes, and 4 rRNA genes.

The phylogenetic analyses were carried out using the complete chloroplast genome sequences of *M. eurybracteata* subsp. *ganpinensis*, 17 reported species of Berberidaceae, and one reported species *Akebia quinata* of Lardizabalaceae as the outgroup. Before constructing the phylogenetic tree, sequence alignment of all the species was performed with the multiple alignment tool MAFFT (Katoh and Standley 2013). With a certain manual correction and gap deletion processing, conserved sequences for constructing phylogenetic analysis were prepared. Maximum Likelihood (ML) tree was constructed using MEGA7.0 (Nguyen et al. 2015). The results showed that *M. eurybracteata* subsp. *ganpinensis* and *M. bealei* were firstly clustered into a branch and the two *Mahonia* species were most closely related to the genus *Berberis* of Berberidaceae. Furthermore, Berberidaceae was grouped into a monophyletic branch and the family could be divided into four groups based on chromosome base number, which was consistent with previous molecular phylogenetic studies on Berberidaceae (Kim, Kim, Kim et al. 2004; Wang et al. 2007; Sun et al. 2018). The herbaceous genera with *x* = 6, *Diphylleia*, *Dysosma*, *Epimedium*, *Sinopodophyllum*, and *Plagiorhegma*, formed into a branch, while *Mahonia* and *Berberis,* which are shrubs or small trees, had chromosome base number with *x* = 7 and were clustered into a branch. The herbaceous genera with *x* = 8, *Gymnospermium* and *Leontice*, formed into a branch and were firstly clustered with the monotypic and shrubby gene *Nandina* with *x* = 10 (Figure 1).

**Figure 1.**
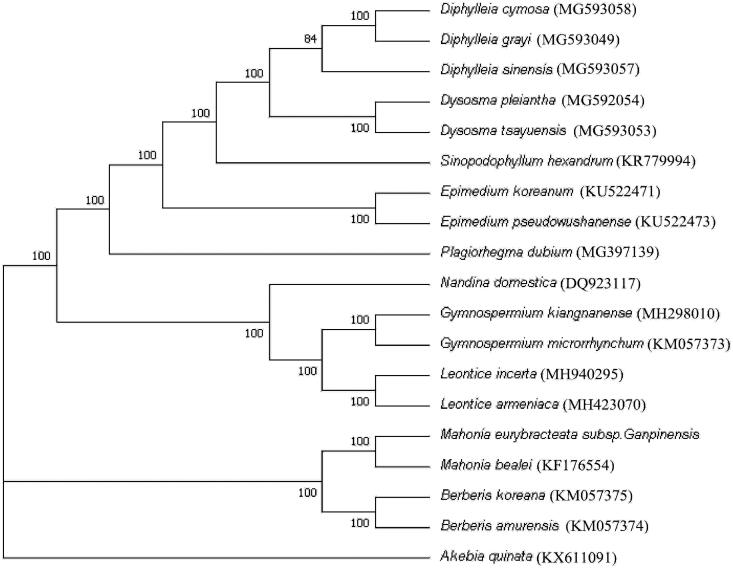
A phylogenetic ML tree constructed using MEGA7.0 based on the complete chloroplast sequence of 19 species including *M. eurybracteata* subsp. *ganpinensis* and *Akebia quinata*.
